# P-548. Higher rate of two-drug regimen discontinuation due to reasons unrelated to virologic failure among people with HIV aged 50 years or above

**DOI:** 10.1093/ofid/ofae631.747

**Published:** 2025-01-29

**Authors:** Tommy Tang, Tsz Shan Kwong, Philip Chan, K W Lam, Helen Chan, Annabel K T Choy, M Y Chu, Jason Fong, Wilson Lam, W S Leung, W M Ting, T C Wu, S K Yung, M P Lee

**Affiliations:** Queen Elizabeth Hospital, Hong Kong SAR, China, Not Applicable, Hong Kong; Queen Elizabeth Hospital, Hong Kong SAR, China, Not Applicable, Hong Kong; Yale University School of Medicine, New Haven, Connecticut; Queen Elizabeth Hospital, Hong Kong SAR, China, Not Applicable, Hong Kong; Queen Elizabeth Hospital, Hong Kong SAR, China, Not Applicable, Hong Kong; Kwong Wah Hospital, Hong Kong, Hong Kong, Hong Kong; Queen Elizabeth Hospital, Hong Kong SAR, China, Not Applicable, Hong Kong; Kwong Wah Hospital, Hong Kong SAR, China, Not Applicable, Hong Kong; Queen Elizabeth Hospital, Hong Kong SAR, China, Not Applicable, Hong Kong; Kwong Wah Hospital, Hong Kong SAR, China, Not Applicable, Hong Kong; Queen Elizabeth Hospital, Hong Kong SAR, China, Not Applicable, Hong Kong; Queen Elizabeth Hospital, Hong Kong SAR, China, Not Applicable, Hong Kong; Queen Elizabeth Hospital, Hong Kong SAR, China, Not Applicable, Hong Kong; Queen Elizabeth Hospital, Hong Kong SAR, China, Not Applicable, Hong Kong

## Abstract

**Background:**

Previous studies demonstrated that integrase strand transfer inhibitor (INSTI)-based two-drug regimens (2DRs) reliably maintain HIV suppression. Corresponding data in older people with HIV (PWH) remain limited. This retrospective study compared the real-world maintenance outcomes of INSTI-based 2DRs between PWH aged 50 or above (PWH >=50) and those below 50 years (PWH< 50). It was approved by the Research and Ethics Committee, Kowloon Central/Kowloon East Cluster, Hospital Authority (KC/KE-23-0195/ER-4).

**Figure:**
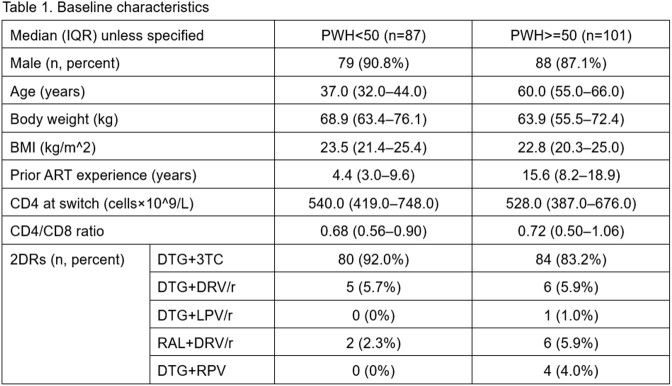

**Methods:**

PWH who switched from suppressive standard three-drug antiretroviral therapy (ART) to oral INSTI-based 2DRs at the HIV Clinic of the Queen Elizabeth Hospital, Hong Kong, China were identified through electronic records. The formulations of 2DRs and outcomes of those who reached 96 weeks after switch, before 1 March 2024 were retrieved. HIV suppression and virologic failure (VF) were defined as plasma HIV-1 RNA < 50 copies/mL and >200 copies/mL respectively.

**Figure:**
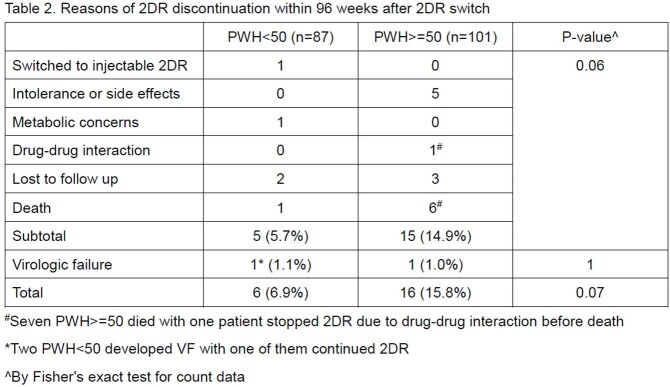

**Results:**

Since January 2014, 237 PWH switched to INSTI-based 2DRs. Their pre-switch characteristics are summarized in **Table 1**. On 29 February 2024, 96 weeks has lapsed from their 2DR switch dates in 87 PWH< 50 and 101 PWH >=50. Eight (4.3%) of them died due to causes unrelated to HIV. Twenty-two of them (11.7%) discontinued 2DRs during the 96-week period: six (6.9%) were PWH< 50 and 16 (15.8%) were PWH >=50, with reasons summarized in **Table 2**.

By log-rank test, PWH >=50 trended toward significance for a higher risk of 2DR discontinuation when compared to PWH< 50 (p=0.058) (**Figure 1**). VF occurred in only 3 (1.6%) users, with similar rates between both groups (p=1). One PWH< 50 developed VF due to non-adherence, continued 2DR and achieved subsequent viral suppression. Another two had negative drug resistance mutation tests afterwards. 2DRs discontinuation due to non-VF causes was numerically higher in the PWH >=50 group without statistical significance (p=0.06).

**Figure 1.**
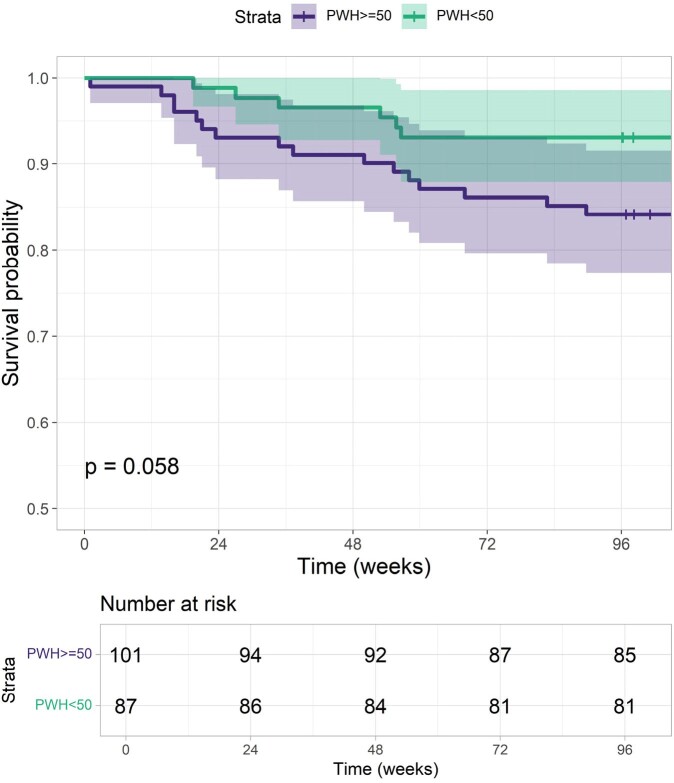
Kaplan-Meier estimator for the cumulative risk of 2DR discontinuation

**Conclusion:**

In this real-world study comparing the maintenance outcomes of virally suppressed PWH who switched to INSTI-based 2DRs, occurrence of VF was uncommon in both older and younger PWH. However, discontinuation due to non-VF causes was more common in older PWH above 50 years old than those below, with a majority contributed by intolerance and non-HIV deaths.

**Disclosures:**

**Wilson Lam, n/a**, Gilead: Advisor/Consultant|Gilead: Honoraria|GSK: Advisor/Consultant|GSK: Honoraria|Moderna: Honoraria|Pfizer: Conference sponsor

